# Evaluation of Asphalt with Different Combinations of Fire Retardants

**DOI:** 10.3390/ma12081283

**Published:** 2019-04-18

**Authors:** Guangji Xu, Xiao Chen, Shichao Zhu, Lingdi Kong, Xiaoming Huang, Jiewen Zhao, Tao Ma

**Affiliations:** 1 School of Transportation, Southeast University, Jiulonghu, Nanjing 211189, China; guangji_xu@seu.edu.cn (G.X.); xche@seu.edu.cn (X.C.); huangxm@seu.edu.cn (X.H.); zhaojiewen@gmail.com (J.Z.); 2 State Engineering Laboratory of Highway Maintenance Technology, Changsha University of Science and Technology, Changsha 410114, China; 3 Qilu Transportation Development Group, 1 Longaoxi, Jinan 200101, China; shichaozhu01@163.com; 4 Shandong Guilu Expressway Construction Co. Ltd., 23 Changrun, Liaocheng 252000, China; mhxkld@163.com

**Keywords:** asphalt, fire retardance, smoke suppression, thermogravimetry, differential thermogravimetry, activation energy, temperature distribution, smoke height distribution

## Abstract

When a fire takes place in a tunnel, the surface of the asphalt pavement will burn and release a large amount of smoke, which is toxic to human health. Thus, in order to prevent the combustion of the asphalt pavement under fire, it is necessary to propose some methods to retard its physical and chemical reaction under the high temperature. In this study, ten different combinations of fire retardants and a control case where no fire retardant was applied were prepared for evaluation. The thermogravimetric (TG)–mass spectrometry (MS) tests were used to evaluate their effect on the fire retardance from mass and energy perspectives and the Fire Dynamics Simulator (FDS) software was used to evaluate the fire retardance from temperature and smoke distribution perspectives. In experimental analysis, the TG (thermogravimetric) and DTG (differential thermogravimetric) curves were used to analyze the mass loss rate and residual mass of the asphalt and the activation energy was calculated and analyzed as well. In addition, decay rate of mass loss rate and increasing rate of activation energy were proposed to evaluate the ease of combustion of the asphalt with and without fire retardants. The results show that in laboratory experiments, the fire retardant combination which includes 48% aluminum hydroxide, 32% magnesium hydroxide, 5% expanded graphite, and 15% encapsulated red phosphorous would lead to an improved effect of fire retardance. In numerical modeling, the temperature and smoke height distribution over time were adopted to evaluate the fire retardance effect. The temperature distribution was found to be symmetrical on both sides of the combustion point and the same combination as proposed in experimental analysis was found to have the best effect on fire retardance due to the largest decrease in temperature. Additionally, because of the highest smoke height distribution, an improved effect on smoke suppression was also found when this combination was applied.

## 1. Introduction

Asphalt binder is a kind of complex mixture composed by macromolecular hydrocarbons and non-hydrocarbons. It contains carbon, hydrogen, and a small number of other elements like sulfur, nitrogen and oxygen [1]. Asphalt mixture is a composite of three-phase materials consisting of aggregates, asphalt binder, and air voids. Under different conditions, the asphalt mixture would represent linear or nonlinear viscoelastic properties, leading to different road performances [2,3,4]. The modification of the asphalt was found to have an impact on the mechanical or rheological properties of the pavement. Karimi (2018) found that activated carbon modification was a robust binder-based conductive component for induced heating and healing of asphalt mixtures [5]. Jahanbakhsh (2016) evaluated the modified asphalt with commonly used modifiers in low temperature performance and found that 0.1 weight% of sulfur could improve thermal cracking resistance [6]. From the existing studies of the asphalt, it can be inferred that the modification could, to some extent, improve the asphalt property, making the asphalt pavement perform better. Normally, the asphalt pavement constructed by base asphalt is easy to combust under fire and will release CO, CO_2_, SO_2_, NO, NO_2_, CH_4_, and other gases, making the environment dangerous for people to survive in due to poisoning or suffocation [7]. Once a fire occurs in a tunnel, it is difficult for people to escape and the difficulty of rescue, evacuation, and fire protection is much higher than that of ordinary roads [8]. Therefore, it is necessary to research the potential modification of asphalt to improve its fire retardance and make it more stable under fire. The study of the fire retardance mechanism of asphalt can fundamentally reduce casualties caused by tunnel fires. Moreover, the impact of recycled asphalt and mixture gradation on the combustion needs further investigation [9,10,11].

Currently, there are many methods that can be used to analyze the combustion process, including experimental and numerical methods. Thermogravimetric (TG)–mass spectrometry (MS) detection is a typical experimental method. It has been applied to many thermal decomposition studies; the material mass change can be monitored and used for building material combustion dynamics models and for speculating the combustion process of the micro-reaction [12,13,14]. Xu et al. (2011) established a multi-order combustion model by using thermogravimetrics and a Fourier infrared spectrometer to study the combustion process [15]. Michelle et al. (2011) studied the thermal degradation parameters of three different asphalt samples and found that one asphalt had the highest activation energy according to the thermogravimetric curve obtained from TG–MS results [16]. 

For numerical analysis, the Fire Dynamics Simulator (FDS) is a computational fluid dynamics (CFD) model which describes the flow of the smoke [17]. It could be used to analyze the performance of the proposed combinations of fire retardants. Temperature and smoke distribution around the fire point could also be obtained to demonstrate the effectiveness of the fire retardants. It solves the Navier–Stokes equations using an explicit finite difference scheme [18]. FDS has been subjected to numerous validation and calibration studies; some cases can be found elsewhere [19]. Currently, FDS has been applied for the performance-based fire safety design, design of smoke control systems, sprinkler/detector activation studies, and so on. M.O. et al. (2013) used FDS to analyze the main feature of fire smoke on human body injury and discussed the impact of smoke and temperature change in the train personnel in emergency evacuation [20]. Kang et al. (2015) adopted FDS to make a risk management of main control board and estimate the time for main control board abandonment [21]. Additionally, Oliveira et al (2019) evaluated how the FDS correlated with experimental fire heights and temperature profiles around a small-scale pool fire and finally provided detailed temperature field around the height of the fire [22].

The aim of this study is to figure out whether the modifiers, i.e., fire retardants, could achieve satisfactory results of fire retardance and evaluate the effect of different modifiers through experimental and numerical analysis. In this study, the base asphalt with ten different fire-retardant combinations and a control case were used to evaluate their fire retardance in both laboratory experiments and numerical modelling. The effects of the different fire-retardant combinations on smoke suppression were also analyzed in numerical modeling.

## 2. Experimental Materials and Methods

### 2.1. Combinations of Fire Retardants and Asphalt

**Figure 1:**
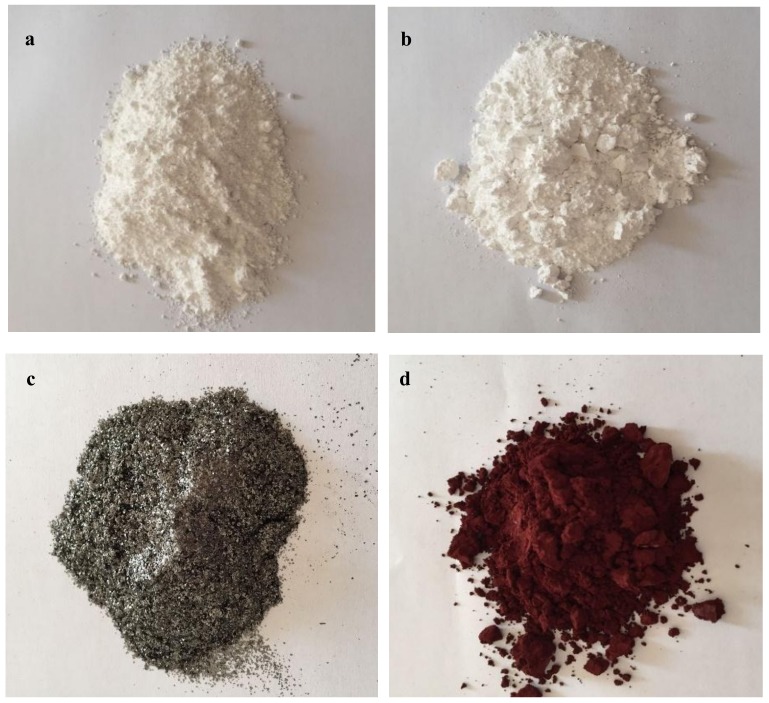
Four fire retardants. (**a**) Aluminum hydroxide; (**b**) magnesium hydroxide; (**c**) expanded graphite; (**d**) encapsulated red phosphorous.

Aluminum hydroxide and magnesium hydroxide are two kinds of widely used fire retardants. Their fire retardance mechanisms are to reduce the combustion temperature by dehydration during the combustion process, thus limiting the combustion speed. Because they can catalyze and oxidize the gas, the amount and the escape rate of the smoke can be reduced [23,24]. Furthermore, the MgO and Al_2_O_3_ generated by aluminum hydroxide and magnesium hydroxide can absorb the smoke and precipitate it into ash [25]. Thus, the aluminum hydroxide and magnesium hydroxide can suppress the smoke during combustion as well. Encapsulated red phosphorous is also an effective fire retardant; it has a strong thermal stability and can reduce the oxygen content on the surface of the combustor, which can limit the occurrence of combustion. Expanded graphite is another effective fire retardant; it can expand and rapidly increase its surface area to form an insulating layer when heated, thus reducing the rate of combustion [26]. Therefore, these four components were selected as basic fire retardants. From the smoke suppression perspective, aluminum hydroxide and magnesium hydroxide were chosen to be the main fire-retardant components. The asphalt used in this study is 70# base asphalt and the appearance of the fire retardants are shown in Figure 1. The related characteristics of fire retardants used in this study are shown in Table 1.

### 2.2. TG–MS Test

The combustion experiments were carried out with a TG–MS system, including a NETZSCH STA 409 thermogravimetric analyzer (TGA) (Bavaria, Germany) and a NETZSCH QMS 403C mass spectrometer (Bavaria, Germany). To start an experiment, a sample of about 10 mg was tiled at the bottom of an Al_2_O_3_ crucible and the internal atmosphere of the TGA was set to simulate the air atmosphere. The N_2_ was set to be 40 mL/min and O_2_ was 10 mL/min. During the test, the temperature remained unchanged at 220 °C. The scanning mode of the mass spectrometry analyzer was ion scanning, which was performed every 105 s. The heating rate was 15 K/min and at least two parallel tests were conducted. The results include TG (thermogravimetric) curves and differential thermogravimetric (DTG) curves, which represent the residual mass of the asphalt and the mass loss rate, respectively. 

### 2.3. Manufacturing Technique and Sample Preparation

First of all, the asphalt was heated to about 180 °C, then different combinations of fire retardants were added into the asphalt equally four times. The mixture was mixed manually until the fire retardants were evenly distributed in the asphalt and no powdery floaters could be seen. The high-speed shear emulsifier was used to ensure the fire retardants were fully distributed in the asphalt. The asphalt was kept to over 100 °C to ensure its rheological property and the initial shear speed was set to be 1000 r/min for 5 min, then increased to 3000 r/min for 10 min, and finally decreased to 1000 r/min for 5 min. After shearing, the modified asphalt was stirred manually for 2 min and was used after natural cooling. 

### 2.4. Evaluation Index in Laboratory Experiments

#### 2.4.1. Thermogravimetric Analysis

Thermogravimetric analysis (TGA) is a method of thermal analysis in which changes in physical and chemical properties of materials are measured as a function of increasing temperature (with constant heating rate) or as a function of time (with constant temperature and/or constant mass loss). It provides a rapid quantitative method to examine the overall combustion process, especially under non-isothermal conditions, and enables one to estimate the effective kinetic parameters for the overall decomposition reactions. The TG and DTG curves obtained from the TG–MS tests were two main methods in TGA. This technique has been widely used in recent years for investigation of combustion or structural characteristics of fossil fuels. The TG curve represents the variation of the residual mass of the asphalt as the temperature increases and a higher residual mass indicates a better fire retardance. DTG is the difference in thermogravimetry ratio of measurement of Dm (weight loss or weight increase) at heating/cooling/isotherm, with interpretation by Dm over temperature or time. In other words, it indicates the variation of mass loss as the temperature increases and the mass loss rate can also be obtained. The mass loss rate represents the efficiency of the mass loss during the combustion process. A higher mass loss rate means that the asphalt is easier to burn. In addition, to compare the difficulty of the asphalt to combust before and after adding fire retardants, decay rate of the maximum mass loss rate was proposed. A higher decay rate of mass loss rate corresponds to a better effect on fire retardance. The definition of the decay rate of mass loss rate is shown in Equation (1).

(1) $$\eta =\frac{{\mathrm{DTG}}_{O}-{\mathrm{DTG}}_{A}}{{\mathrm{DTG}}_{O}}\times 100\%,$$

where DTG_O_ = maximum mass loss rate of asphalt during combustion without fire retardant (%/min); DTG_A_ = maximum mass loss rate of asphalt during combustion or pyrolysis with fire retardant (%/min); η = decay rate of mass loss rate (%).

#### 2.4.2. Growth Rate of Activation Energy

Activation energy is an internal factor that determines the rate of the chemical reaction; it can be used to evaluate the ease of combustion [27]. The higher the activation energy is, the more energy is needed for the combustion of the material and vice versa. In this study, the Coats–Redfern integral method was adopted to calculate the activation energy, which has been applied elsewhere [28]. In addition, the growth rate of activation energy was proposed in this study to evaluate the ease of combustion before and after adding fire retardants. The definition of activation energy is shown in Equation (2).

(2) $$\lambda =\frac{{\mathrm{AE}}_{A}-{\mathrm{AE}}_{O}}{{\mathrm{AE}}_{O}}\times 100\%,$$

where AE_O_ = activation energy of asphalt during combustion without fire retardant (KJ/mol); AE_A_ = activation energy of asphalt during combustion with fire retardant (KJ/mol); λ = growth rate of activation energy. 

### 2.5. Thermodynamic Properties of Four Basic Fire Retardants

**Figure 2:**
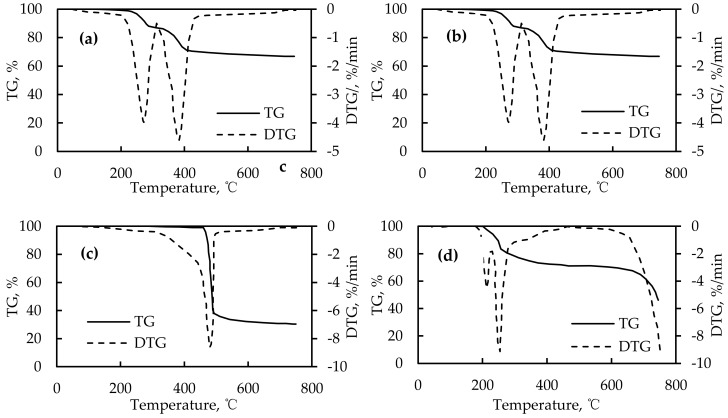
Thermogravimetric–differential thermogravimetric (TG–DTG) curves of four fire retardants. (**a**) Aluminum hydroxide; (**b**) magnesium hydroxide; (**c**) encapsulated red phosphorous; (**d**) expanded graphite.

The thermodynamic properties of basic fire-retardant components may be different, therefore, it is necessary to analyze their thermodynamic behaviors accurately to determine the appropriate ratio of each component. The thermodynamic characteristics of the four basic fire retardants were studied in order to evaluate their fire retardance effects and provide a theoretical basis for the design of fire retardant combinations. Figure 2 shows the TG and DTG curves of the four basic fire retardants.

As shown in Figure 2, the aluminum hydroxide had two peak temperatures: 269 °C and 383 °C, respectively. Its peak thermal weight loss rate was only −4% per minute, while its duration was long and the operating temperature ranged from 200 to 480 °C. It can be inferred that aluminum hydroxide is a relatively long-lasting fire retardant, but its fire retardance efficiency is relatively low. Thus, its proportion in the fire-retardant combination should be relatively high in order to improve the fire retardance efficiency. Magnesium hydroxide had a peak temperature of 285 °C and a peak thermal weight loss efficiency of −8% per minute. However, its duration was short and its working temperature ranged from 200 to 350 °C. It can be inferred that magnesium hydroxide is not a very lasting fire retardant, but its fire retardance efficiency is high. Thus, its content should be relatively low in the fire-retardant combination. With regard to the encapsulated red phosphorous, it had a peak temperature of 495 °C and its peak thermal weight loss efficiency was −8% per minute. However, its duration was very short and its working temperature ranged from 450 to 500 °C. It can be inferred that the fire retardance effect of encapsulated red phosphorus only works when the asphalt component exhibits a relatively high peak temperature. Additionally, there were two peak temperatures of expanded graphite at an early stage of the combustion, which were 212 °C and 254 °C, respectively. Its peak thermal weight loss efficiency was −8% per minute. Because the expanded graphite is primarily used for heat insulation, its chemical reaction should precede that of other fire retardants to effectively form a heat insulation layer. Therefore, its dosage should be strictly controlled to avoid affecting the road performance because it would cause great expansion when heated.

### 2.6. Experiment Design

Given that the expanded graphite would expand greatly after heating, some preliminary experiments were conducted to determine the appropriate ratio of basic fire retardants. According to the test results, the dosage of the expanded graphite was limited to 5% of the total mass of the fire retardants and the best ratio of the encapsulated red phosphorous to the expanded graphite was decided to be 3:1. Table 2 shows the different combinations of fire retardants.

## 3. Numerical Analysis Model and Methods

### 3.1. Model Establishment

**Figure 3:**
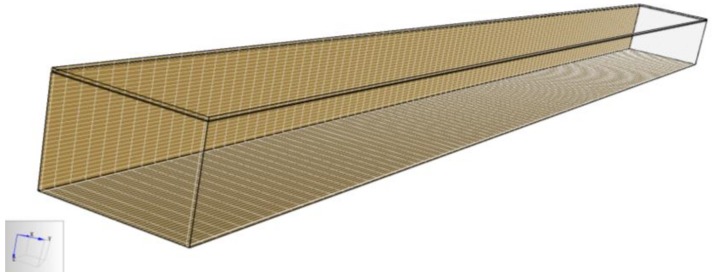
Numerical model and grid generation of the tunnel.

In this study, FDS was adopted to simultaneously calculate the temperature and smoke field in a fire. The size of the tunnel model in this study was 100 m long, 10 m wide, and 7.2 m high. 20-cm thick concrete was built in the tunnel and the pavement at the bottom of the tunnel was divided into three layers. Two asphalt surface layers were, respectively, 4 cm and 6 cm from top to bottom and the third concrete layer was set to be 10-cm thick. Figure 3 shows the numerical model and its grid division. 

According to the model size, the grid was divided into 100 grids (1 m for each grid) in the longitudinal direction of the tunnel, 40 grids (0.25 m for each grid) in the transverse section, and 28 grids (0.26 m for each grid) in the vertical direction. Two kinds of materials, which were, respectively, cement concrete and asphalt concrete, were created in the model. The surface parameters of the fire source, asphalt concrete, and cement concrete were established in the “SURFACE” module. A tunnel model was established by the “OBSTRUCTURE” module and a fire source (2 m × 2 m on the road surface at the center of the tunnel), two asphalt pavement layers, a cement concrete layer, and vents on both sides of the tunnel were set in the “VENT” module in FDS. Observation points were created at different horizontal and vertical distances from the fire source to record the distribution of the temperature and smoke during the combustion process. Finally, the running time of FDS was selected to be 100 s in this study. 

The temperature and smoke height distributions over time were recorded. The monitor points of the temperature were along the transverse and longitudinal directions of the tunnel; 9 of them were along the transverse direction and 6 of them were along the longitudinal direction. For the smoke height, a total of 6 monitor points was set up along the longitudinal direction of the tunnel.

### 3.2. Evaluation Index in Numerical Analysis

In the numerical modeling, the distribution of the temperature field after combustion can directly reflect the effect of fire retardance. The rate of increase in temperature would be reduced due to the fire retardants, which would in turn affect the distribution of the temperature field. In addition, the height distribution of the smoke can also be obtained in simulation; it is an important index for evaluating the survival probability of disaster victims [29,30] and is also an important parameter for analyzing the smoke suppression effect of fire retardants. As the smoke height decreased, its density correspondingly decreases, making the smoke flow upward. Therefore, the higher the smoke distributes, the better the smoke is suppressed. Furthermore, the cloud charts in FDS could be used to show the smoke height distribution over time [31]. Therefore, the temperature and smoke height distribution were used to evaluate the fire retardance and smoke suppression in the numerical analysis. 

## 4. Results

### 4.1. Experimental Analysis of Fire Retardance Effects Based on the TG Curve

**Figure 4:**
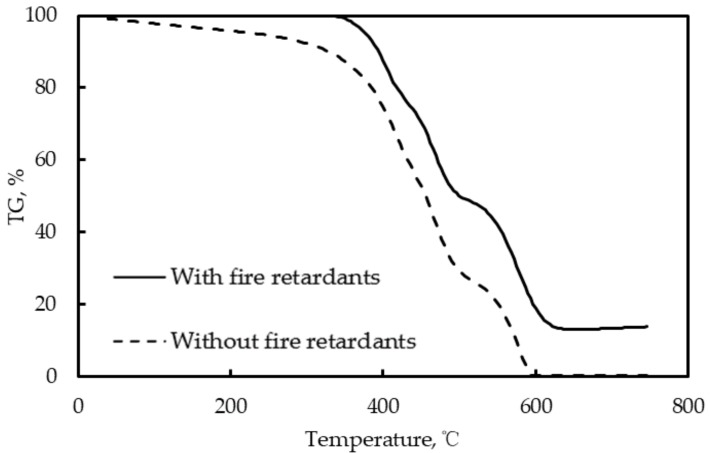
Typical TG curve of the asphalt with or without fire retardant.

Figure 4 shows a typical TG curve of the asphalt with the combination of fire retardants. From Figure 4, it can be noted that the fire retardants do have an effect on retarding combustion due to the reduced mass loss. Compared to the control case, the temperature for the asphalt with fire retardants increased more than 300 °C before dehydration, which can ensure the asphalt a stable status under the high temperature. Thus, the fire retardants can help the asphalt maintain the mass. The residual mass percentage is listed in Table 3. From the results of combination 1 and combination 6, it can be found that the aluminum hydroxide solely had a better fire retardance than magnesium hydroxide because of its higher residual mass percentage. Furthermore, adjusting the basic fire-retardant content in the asphalt only leads to a little variation in fire retardance and no remarkable trend or relationship is found. Among all the tests, combination 9, which included 48% aluminum hydroxide, 32% magnesium hydroxide, 5% expanded graphite, and 15% encapsulated red phosphorous, results in the best fire retardance and combination 5, which included 80% aluminum hydroxide and 20% magnesium hydroxide leads to the least remarkable effect.

### 4.2. Experimental Analysis of Fire Retardance Effects Based On the DTG Curve

**Figure 5:**
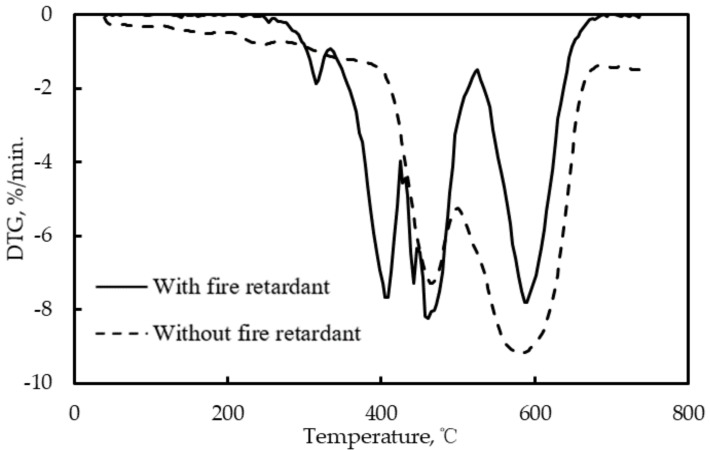
Typical DTG Curve of the Asphalt.

Figure 5 shows the typical DTG (differential thermogravimetric) curve of the asphalt with the combination of fire retardants. As seen, the largest mass loss rate of the asphalt without the fire retardants was larger than that with fire retardants, indicating that the fire retardants can retard the asphalt combustion and in turn retard the mass loss. Table 4 shows the maximum mass loss rate and the decay rate of mass loss rate of different cases. It can be found that the variation in the basic fire-retardant content only leads to a little change in the two indexes and the aluminum hydroxide indicated a better fire retardance than magnesium hydroxide due to its lower maximum mass loss rate and higher decay rate of mass loss rate, which are similar to the results in TG curve analysis. Among all the cases, combination 9 shows the lowest mass loss rate and the highest decay rate of mass loss rate, indicating the best fire retardance effect. This result is the same as the analysis based on the TG curves. However, the combination which leads to the least remarkable fire retardance differs from that in the analysis based on the TG curves.

### 4.3. Experimental Analysis of Fire Retardance Effects Based on Activation Energy

Table 5 shows the activation energy and the corresponding increasing rate of each combination of fire retardants. As seen, the aluminum hydroxide had a higher activation energy and increasing rate than magnesium hydroxide, indicating a better fire retardance effect. Combination 9 shows the highest activation energy and increasing rate, which means the asphalt is the most difficult to burn when these fire retardants are added. This result correlates well with the analysis based on the TG and DTG curves. However, the lowest activation energy and increasing rate were found in combination 1, so this combination may have the least remarkable effect on fire retardance, which is different from neither TG analysis nor DTG analysis. Thus, further research is needed.

### 4.4. Numerical Analysis of Fire Retardance Effects Based on Temperature Distribution over Time

**Figure 6:**
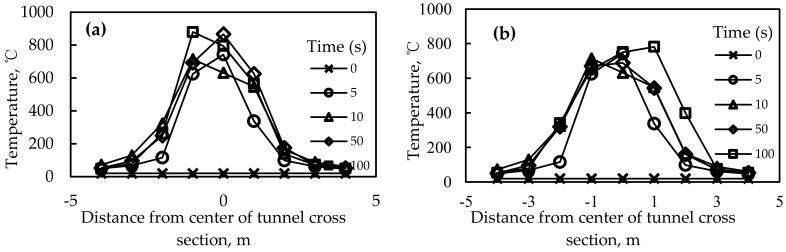
Distribution of temperature along the transverse direction; (**a**) without fire retardant; (**b**) with fire retardant.

Figure 6 shows the distribution of the temperature along the transverse direction at the combustion point (0 m, 1 m, 2 m, 3 m, 4 m in the transverse direction of the combustion point) when no fire retardant was added and Figure 6b shows a typical distribution of the temperature along the transverse direction at the combustion point when fire retardants were added. It can be found that the temperature approximately presented a symmetrical distribution from the center of the combustion point. In the control case where no fire retardant was applied, the maximum temperature at each time point increased as the temperature increased and the highest temperature reached nearly 900 °C. However, after the addition of fire retardants, the temperature at all points decreased to different degrees and the decrease in the maximum temperature of the asphalt with fire-retardant combination 9 was only 759 °C, which fully demonstrated that the addition of fire retardants effectively inhibited the increase of temperature. Also, the asphalt with fire-retardant combination 6 exhibited a lower temperature at all points from the combustion point than combination 1, indicating that the aluminum hydroxide had a better effect on fire retardance than magnesium hydroxide.

**Figure 7:**
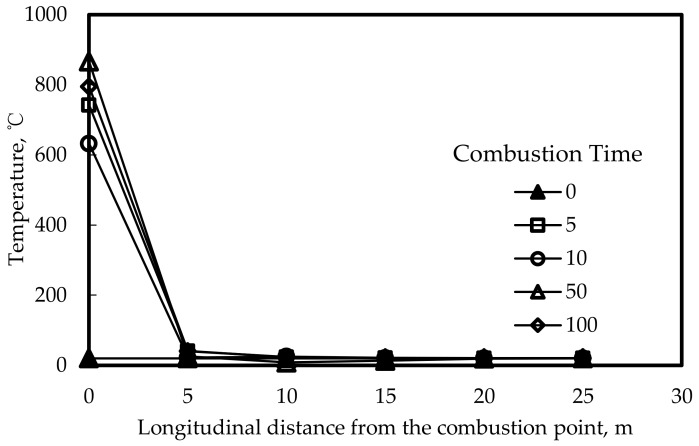
Distribution of the temperature along the longitudinal direction.

Figure 7 shows a typical distribution of the temperature at the combustion point (0 m, 5 m, 10 m, 15 m, 20 m, 25 m in the longitudinal direction of the combustion point). It can be found that the temperature increased with time and the temperatures were all lower than 50 °C when it was 5 m away from the combustion point, indicating that the affected distance of the combustion along the longitudinal direction was less than 5 m.

### 4.5. Numerical Analysis of Fire Retardance Effects Based on the Smoke Height Distribution over Time

**Figure 8:**
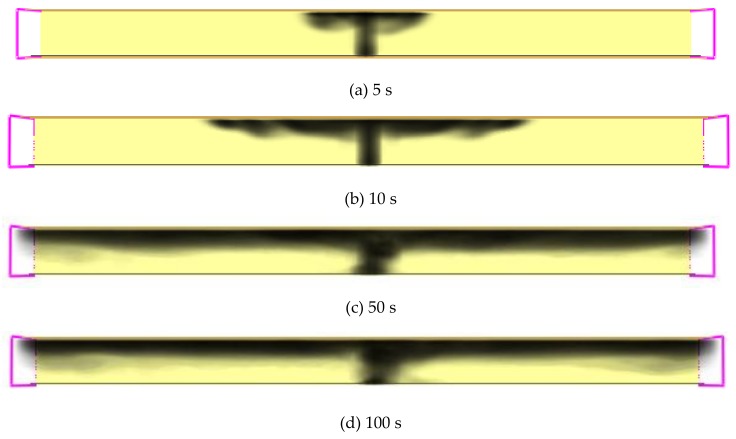
Smoke height distribution over time without fire retardant additive.

Figure 8 shows the smoke height distribution over time when no fire retardant was added. Due to the restriction of the software function in the current version, only the smoke cloud chart without a legend could be obtained and many other researchers had used it to achieve satisfactory results [31,32]. Its distribution was analyzed to evaluate the effect on smoke suppression.

**Figure 9:**
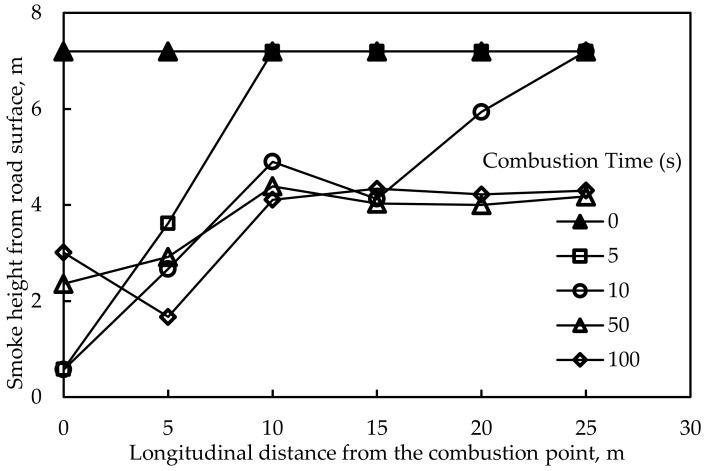
Smoke height distribution along the longitudinal direction (without fire retardant).

It can be seen that after the combustion, the smoke gradually expanded to the exits on both sides of the tunne and the height of the smoke in the tunnel decreased continuously. Due to the limitation of paper space, the smoke height distribution in the following analysis was reflected by smoke height distribution instead of cloud charts. Figure 9 shows the smoke height distribution along the longitudinal direction at the combustion point when no fire retardant was added. It shows that the smoke height was 2 m when it was 5 m away from the combustion point. Given that the height of people is normally under 2 m, thus, people in this range would be poisoned by the smoke in the tunnel.

**Figure 10:**
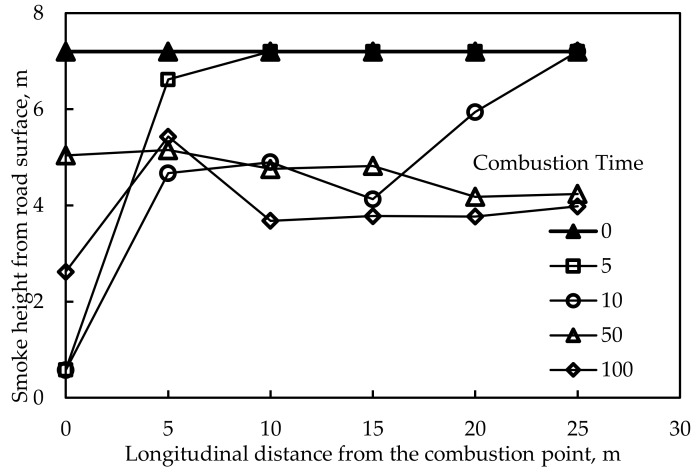
Smoke height distribution along the longitudinal direction (with fire retardants).

Figure 10 is a typical smoke height distribution along the longitudinal direction. Among them, the smoke height increased to different degrees when it was 5 m away from the combustion point and combinations 1 and 9 whose smoke height were the highest led to the best effect on smoke suppression. Taking the temperature distribution into consideration, the combination of fire retardants which would lead to optimal fire retardance and smoke suppression was combination 9. It is also consistent with the test results in laboratory experiments.

## 5. Conclusions

In this study, in order to research the effect of different modifiers on fire retardance, various combinations of fire retardants were added into the base asphalt and analyzed by laboratory experiments and numerical modeling. The following conclusions were drawn:

1. Among all the combinations of fire retardants used in this study, the combination of 48% aluminum hydroxide, 32% magnesium hydroxide, 5% expanded graphite, and 15% encapsulated red phosphorous leads to the best effect on fire retardance in experimental analysis. In numerical modeling, that combination also leads to an improved smoke suppression effect, while further research is needed to evaluate it in the real scenario.
2. The aluminum hydroxide indicated a better effect on fire retardance and smoke suppression than the magnesium hydroxide in both experimental and numerical analysis.
3. The temperature distribution on both sides of the combustion point is basically symmetrical. When the fire retardants were added, the temperature at each time and space point decreased to different degrees. Among all the combinations adopted in this study, it led to the largest decrease when 48% aluminum hydroxide, 32% magnesium hydroxide, 5% expanded graphite, and 15% encapsulated red phosphorous were added into the asphalt.
4. When no fire retardant was added, the smoke height at 5 m away from the combustion point was about 2 m. Among all the combinations adopted in this study, when fire retardants were added, the smoke height increased to different degrees and the height distribution became the highest when 48% aluminum hydroxide, 32% magnesium hydroxide, 5% expanded graphite, and 15% encapsulated red phosphorous were added.

## Figures and Tables

**Table 1:** Fire retardant characteristics.

	Aluminum Hydroxide	Magnesium Hydroxide	Expanded Graphite	Encapsulated Red Phosphorous
Moisture Content	0.15%	0.3%	0.1%	0.65%
Mean Size	20 nm	1.2 μm	7.5 μm	12.5 μm
Density	0.15 g/cm^3^	2.36 g/cm^3^	2.1 g/cm^3^	2.2 g/cm^3^
Purity	99.9%	96.0%	99.0%	70%
Specific Surface Area	5 m^2^/g	200 m^2^/g	150 m^2^/g	-

**Table 2:** Fire-retardant combinations.

Combination Number	Aluminum Hydroxide (%)	Magnesium Hydroxide (%)	Expanded Graphite (%)	Encapsulated Red Phosphorous (%)
1	0	100	0	0
2	20	80	0	0
3	40	60	0	0
4	60	40	0	0
5	80	20	0	0
6	100	0	0	0
7	16	64	5	15
8	32	48	5	15
9	48	32	5	15
10	64	16	5	15
11	0	0	0	0

Note: The mass ratio of the fire retardants is determined to be 10% of the asphalt.

**Table 3:** Residual mass results of the asphalt.

Combination Number	Aluminum Hydroxide (%)	Magnesium Hydroxide (%)	Expanded Graphite (%)	Encapsulated Red Phosphorous (%)	Residual Mass Percentage (%)
1	0	100	0	0	14
2	20	80	0	0	16
3	40	60	0	0	15
4	60	40	0	0	20
5	80	20	0	0	12
6	100	0	0	0	17
7	16	64	5	15	17
8	32	48	5	15	19
9	48	32	5	15	23
10	64	16	5	15	19
11	0	0	0	0	0

**Table 4:** Decay rate of mass loss rate of the asphalt.

Combination Number	Maximum Mass Loss Rate (%/min.)	Decay Rate Of Mass Loss Rate (%)
1	−7.9	13.4
2	−7.9	13.4
3	−8.0	12.3
4	−7.4	18.9
5	−7.9	13.4
6	−7.8	14.5
7	−7.8	14.5
8	−7.5	17.8
9	−7.2	21.1
10	−7.8	14.5
11	−9.2	-

**Table 5:** Activation energy and its increasing rate of the asphalt.

Combination Number	Activation Energy (kJ/mol)	Increasing Rate of Activation Energy (%)
1	117.12	5.17
2	124.8	12.07
3	126.72	13.79
4	128.64	15.52
5	120.96	8.62
6	126.72	13.79
7	124.8	12.07
8	126.72	13.79
9	132.48	18.97
10	120.96	8.62
11	103.62	3.21
